# Long-Term Illness in Adults Hospitalized for Respiratory Syncytial Virus Disease, United States, February 2022–September 2023

**DOI:** 10.3201/eid3114.241982

**Published:** 2025-12

**Authors:** Aleda M. Leis, Kelsey N. Womack, Courtney Maxcy, Ellen Caldwell, Caroline Cheng, Sydney A. Cornelison, Diya Surie, Fatimah S. Dawood, Sharon Saydah, Manjusha Gaglani, Cristie Columbus, Abhijit Duggal, Laurence W. Busse, Laurynn M. Giles, Ivana A. Vaughn, Ithan D. Peltan, David N. Hager, Amira Mohamed, Matthew C. Exline, Akram Khan, Jennifer G. Wilson, Jarrod S. Mosier, Steven Y. Chang, Adit A. Ginde, Nicholas M. Mohr, Christopher Mallow, Estelle S. Harris, Nicholas J. Johnson, Kevin W. Gibbs, Jennie H. Kwon, Basmah Safdar, Emily T. Martin, Wesley H. Self, Catherine L. Hough, Jin H. Han, for the Investigating Respiratory Viruses in the Acutely Ill (IVY) Network

**Affiliations:** University of Michigan School of Public Health, Ann Arbor, Michigan, USA (A.M. Leis, C. Cheng, E.T. Martin); Vanderbilt Institute for Clinical and Translational Research, Vanderbilt University Medical Center, Nashville, Tennessee, USA (K.N. Womack, W.H. Self); Oregon Health & Sciences University, Portland, Oregon, USA (C. Maxcy, E. Caldwell, A. Khan, C.L. Hough); Vanderbilt University Medical Center, Nashville (S.A. Cornelison, J.H. Han); Centers for Disease Control and Prevention, Atlanta, Georgia, USA (D. Surie, F.S. Dawood, S. Saydah); Baylor College of Medicine–Temple, Temple, Texas, USA (M. Gaglani); Baylor Scott & White Health, Dallas, Texas, USA (M. Gaglani, C. Columbus); Texas A&M University College of Medicine, Dallas (M. Gaglani, C. Columbus); Cleveland Clinic, Cleveland, Ohio, USA (A. Duggal); Emory University, Atlanta (L.W. Busse); Hennepin County Medical Center, Minneapolis, Minnesota, USA (L.M. Giles); Henry Ford Medical Center, Detroit, Michigan, USA (I.A. Vaughn); Intermountain Medical Center, Murray, Utah, USA (I.D. Peltan); University of Utah, Salt Lake City, Utah, USA (I.D. Peltan, E.S. Harris); Johns Hopkins University School of Medicine, Baltimore, Maryland, USA (D.N. Hager); Montefiore Medical Center, Albert Einstein College of Medicine, Bronx, New York, USA (A. Mohamed); The Ohio State University, Columbus, Ohio, USA (M.C. Exline); Stanford University School of Medicine, Stanford, California, USA (J.G. Wilson); University of Arizona, Tucson, Arizona, USA (J.S. Mosier); University of California, Los Angeles, California, USA (S.Y. Chang); University of Colorado School of Medicine, Aurora, Colorado, USA (A.A. Ginde); University of Iowa, Iowa City, Iowa, USA (N.M. Mohr); University of Miami, Miami, Florida, USA (C. Mallow); University of Washington, Seattle, Washington, USA (N.J. Johnson); Wake Forest School of Medicine, Winston-Salem, North Carolina, USA (K.W. Gibbs); Washington University, St. Louis, Missouri, USA (J.H. Kwon); Yale University, New Haven, Connecticut, USA (B. Safdar); Geriatric Research, Education, and Clinical Center, Tennessee Valley Healthcare System, Nashville (J.H. Han)

**Keywords:** respiratory syncytial virus, RSV, viruses, respiratory infections, COVID-19, SARS-CoV-2, United States

## Abstract

Respiratory syncytial virus (RSV) can cause severe illness, but little is known about long-term consequences in hospitalized adults. We surveyed adults (>18 years of age) who survived hospitalization for RSV or COVID-19 during February 2022–September 2023 about physical functioning and quality of life; surveys were conducted 6–12 months after hospitalization. We compared outcomes after RSV hospitalization by age (<60 vs. >60 years) and to those hospitalized for COVID-19 by using multivariable regression models. Among 146 adults hospitalized with RSV, 27.4% reported severe breathlessness and 21.9% poor quality of life at follow-up. Few differences were seen in posthospital illness by age. After adjustment, participants with RSV had 1.81 (95% CI 1.08–3.04) times increased odds of worse dyspnea than did those with COVID-19. Participants reported functional and quality of life impairments after RSV hospitalization, regardless of age, and a postdischarge sequelae constellation similar to that for those hospitalized for COVID-19.

Respiratory syncytial virus (RSV) can cause severe acute respiratory illness, especially in older adults. In the United States, RSV infections are responsible for ≈100,000–150,000 hospitalizations annually in persons >60 years of age (*1*) and an estimated economic burden of >$1.5 billion annually (*2*). RSV infections in older adults comprise 11% of hospital admissions for pneumonia and chronic obstructive pulmonary disease, 7% for acute heart failure, and 5% for asthma exacerbation (*3*). Compared with adults hospitalized and vaccinated for influenza and COVID-19 in 2022–23, those hospitalized with RSV were more likely to be in the intensive care unit (ICU) and receive mechanical ventilation and are at higher risk for death in the hospital and by 1 year after hospitalization (*4*–*6*).

The COVID-19 pandemic highlighted the long-term sequelae associated with severe acute respiratory viral infection. Survivors of moderate-to-severe COVID-19 have substantial and persistent impairments in cognitive and physical function and mental health, leading to loss of independence and health-related quality of life (*7*–*9*) similar to those after other critical illnesses (*10*–*13*). Less is known about recovery after hospitalization with RSV. A previous report found that 10% of adults hospitalized with RSV continue to have moderate-to-severe dyspnea, fatigue, or sleep disruption 3 months after discharge (*14*). One quarter of patients >60 years of age may have worsening dyspnea, and one third experience worsening functional impairment 6 months after hospitalization (*15*). However, those studies were limited by narrow capture of outcomes (*14*), focusing only on older cohorts (*15*), and limiting follow-up to 6 months (*14*,*15*).

Substantial gaps remain in determining the long-term consequences of hospitalization with RSV. The primary objective of the prospective multicenter Surveillance of Respiratory Infections’ Sequelae (SunRISE) program is to describe posthospital functional, physical, symptom, and quality of life outcomes of patients hospitalized with acute RSV and other acute respiratory infections (ARI) up to 1 year after index hospitalization. The objectives of this analysis are to characterize patients in the SunRISE program and describe the burden of physical disability, loss of independence, persistent symptoms, and poor quality of life outcomes 6–12 months after hospitalization with RSV; examine those outcomes after hospitalization for RSV among adults >60 years versus those <60 years of age, given RSV vaccination recommendations for those >60 years of age starting in June 2023 (*16*); and compare 6–12-month outcomes to outcomes among persons hospitalized with COVID-19.

## Methods

### Study Design and Setting

SunRISE is a nested posthospitalization surveillance project involving adult patients hospitalized in 26 hospitals in 20 US states participating in the Investigating Respiratory Viruses in the Acutely Ill (IVY) Network, funded by the Centers for Disease Control and Prevention. Full IVY methods can be found elsewhere (*17*–*19*). In brief, IVY enrolls adults (>18 years of age) admitted to participating hospitals with a clinical syndrome consistent with acute respiratory illness (>1 signs and symptoms of fever, cough, shortness of breath, hypoxemia, or new pulmonary findings on chest imaging consistent with pneumonia). Enrolled participants are systematically tested for RSV, SARS-CoV-2, and influenza by laboratory-based PCR on a nasal swab sample within 10 days of symptom onset (*17*–*19*). 

### Selection of Participants

Patients were eligible for SunRISE if they survived to hospital discharge. Patients were ineligible if the patient or their surrogate was unable to communicate in English or Spanish or if they had no reliable telephone access. This analysis includes patients enrolled in SunRISE after hospitalization with either RSV (primary analytic cohort) or COVID-19 (comparator cohort) during February 2022–September 2023. For this analysis, we excluded patients who tested positive for multiple viruses (RSV, SARS-CoV-2, or influenza) during the index hospitalization and those in hospice care at hospital admission or discharge.

**Figure 1:**
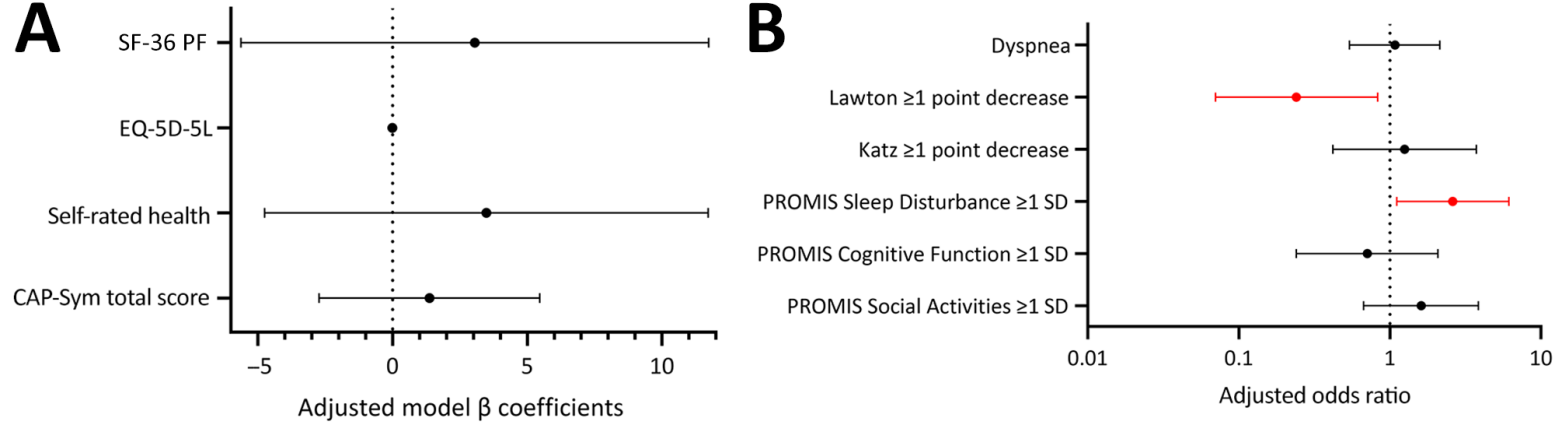
Multivariable model results for 6- to 12-month primary and secondary outcomes for patients hospitalized with respiratory syncytial virus by age in a study of long-term illness in adults hospitalized for respiratory syncytial virus disease or COVID-19, United States, February 2022–September 2023. Models compared persons <60 years of age to those >60 years of age. Results are presented separately for continuous (A) and binary or ordinal (dyspnea) (B) outcomes. The earliest completed survey from 6, 9, or 12 months was included. Models were additionally adjusted for sex, race/ethnicity, smoking status, baseline functional limitations, and number of organ systems affected by chronic disease. For outcome models with baseline data available (Katz, Lawton, and SF-36 PF), the matching retrospective baseline variable was included. Red indicates statistically significant effects. Error bars indicate 95% CIs. Vertical dotted lines indicate a null result value for that model type. Outcomes where higher values indicate worse illness for those <60 years of age: CAP-Sym total score, dyspnea, Lawton >1 point decrease, Katz >1 point decrease, and PROMIS Sleep Disturbance >1 SD. Details on each testing scale are provided in the text.

SunRISE telephone survey attempts began on April 26, 2023, and contact occurred up to 14 months after discharge. We recontacted patients who were still in the hospital at the time of initial contact after discharge. Eligible patients were approached at 6, 9, or 12 months after hospital admission at the earliest survey window in which they were eligible (Appendix 2 Figure 1), enabling entry at any follow-up time point; we recontacted participants who were unable to be reached at the next open survey window. We prioritized patients hospitalized with RSV for contact because the primary goal was to study RSV-related sequelae. We matched hospitalized RSV patients with COVID-19 patients 1:1 by admission date (within 30 days where possible) and site; not all RSV participants were matched (n = 1 in current analysis). We approached both IVY participants and proxies; whenever possible, we prioritized collecting data directly from the participant. To maximize survey completion, we offered surveys by telephone, email link, or postal mail, based on respondent preference; we offered a Spanish-language version of the survey in all forms. We collected data using Research Electronic Data Capture (REDCap) (*20*,*21*). If the patient had multiple surveys completed (e.g., 6 and 9 months), we used the earliest follow-up timepoint with completed data because most participants had 6-month data.

### Primary Outcomes

The primary outcomes for this analysis were physical function; degree of dyspnea; ability to perform basic activities of daily living (ADLs), such as bathing, feeding, and dressing, and instrumental ADLs, such as shopping, managing finances, or making telephone calls; self-rated health; and quality of life (Appendix 2 Table 1). We characterized physical function by using the physical functioning subscale of the RAND 36-Item Health Survey 1.0 (SF-36 Physical Functional Subscale), which ranges from 0 to 100, with higher scores indicating better physical functioning (*22*); its overall population mean (+SD) is 70.6 (+27.4) (*23*). At the first survey contact, we asked patients to rate physical function 2 weeks before the acute illness using the SF-36 Physical Function Subscale. We characterized the degree of dyspnea using a modified Medical Research Council (MRC) dyspnea scale (*24*), for which scores range from grade 0 (only gets breathless during strenuous exercise) to grade 4 (gets too breathless to leave the house).

The Katz Index of Independence in ADLs characterized the patient’s ability to perform basic ADLs (*25*). Scores range from 0 to 6, and higher scores indicate greater independence in performing basic ADLs (*25*). The Lawton Instrumental Activities of Daily Living characterized the patient’s ability to perform instrumental ADLs (*26*). Scores range from 0 to 8, and higher scores indicate greater independence in performing instrumental ADLs (*26*). At the first contact, we asked patients to rate their ability to perform their basic and instrumental ADLs 2 weeks before the acute illness, using both scales to establish a baseline. We considered a decrease of >1 point compared with baseline on the Katz or Lawton scale a loss of 1 basic or instrumental ADL, respectively.

We assessed quality of life by using the EuroQol 5-dimension, 5-level questionnaire (EQ-5D-5L), which uses 5 questions to characterize impairments in mobility, self-care, usual activities, pain/discomfort, and anxiety/depression to calculate utility index values validated for the US population (*27*). A utility index value of 1.0 indicates perfect health, 0.0 represents death, and <0.0 indicates quality of life worse than death. The EQ-5D-5L’s population mean (+SD) is 0.85 (+0.21) (*28*). An EQ-5D-5L score <0.632 indicates fair quality of life, and a score <0.338 indicates poor quality of life (*28*). We also asked patients to report self-rated health on a scale from 0 (the worst health) to 100 (the best health). The self-rated health rating’s overall population mean (+SD) is 80.4 (+15.6) (*28*).

### Secondary and Exploratory Outcomes

We characterized sleep, cognition, and involvement with social activities by using the Patient-Reported Outcomes Measurement Information System (PROMIS) Sleep Disturbance (4 items) (*29*,*30*), Cognitive Function Abilities Subset (6 items) (*31*), and Social Roles and Activities (4 items) (*32*), respectively. We scored PROMIS scales by using the online HealthMeasures service, with default populations selected. The population means for all PROMIS scales are standardized to 50 (SD +10). Higher scores represent poorer sleep quality for the PROMIS Sleep Disturbance, and lower scores represent worse cognitive functioning for the PROMIS Cognitive Function Abilities subset and decreased involvement in social activities for the Social Roles and Activities subset. We assessed symptoms using a modified 15-item version of the Community-Acquired Pneumonia Symptoms Survey (*33*) (CAP-Sym), scored 0–75, with each symptom rated on a scale of 0 (no symptom presence) to 5 (severe) and higher scores indicating greater symptom severity burden. Participants with missing data for a given symptom in the CAP-Sym score were imputed to not have that symptom. Symptoms were severe if rated 4 or 5 in severity.

We also assessed exploratory outcomes. Those outcomes included the use of home help and living in a long-term care facility or skilled nursing facility (LTCF/SNF), 2 items about missed work or school for patients and caregivers, new or worsened home oxygen use, and new or worsened continuous positive airway pressure or other breathing machine use compared with 1 month before hospitalization.

### Data Collected during Acute Hospitalization

Data collected from the index hospitalization included ICU admission; discharge location, such as home or skilled nursing facility; and length of stay. Severe in-hospital outcomes were any of the following events: deep vein thrombosis, pulmonary embolism, myocardial infarction, stroke, high-flow nasal cannula oxygen, noninvasive or invasive mechanical ventilation, new tracheostomy, new renal replacement therapy, use of vasopressors, or use of extracorporeal membrane oxygenation.

### Statistical Analysis

We computed descriptive statistics, including frequencies and percentages, means with SDs, or medians with interquartile ranges (IQR), for demographic data and outcome variables. We calculated p values for comparisons between adults <60 years versus >60 years of age and between patients with RSV versus those with COVID-19 by using χ^2^ or Fisher exact tests for categorical variables and independent *t* or Wilcoxon signed-rank tests for continuous variables, as appropriate.

We constructed multivariable regression models separately for each primary and secondary outcome of interest to compare age <60 years versus >60 years among patients hospitalized with RSV, as well as to compare patients hospitalized with RSV to those hospitalized with COVID-19, adjusting for potential confounders. Model forms included linear, logistic, and ordinal regression, as appropriate; model construction was based on sample size and number of outcomes to avoid the overfitting of models. We used firth correction for rare outcomes, where appropriate. We adjusted all models for age, sex, race and ethnicity, smoking status at index hospitalization, number of organ systems affected by chronic disease, and baseline physical functioning limitations (defined as requiring home care help or unable to walk independently) and additionally adjusted dyspnea models for chronic pulmonary disease as a sensitivity analysis. Models comparing persons hospitalized with RSV to those hospitalized with COVID-19 included a virus variable. For outcome models with baseline Katz, Lawton, and SF-36 Physical Functional Subscale data available, we included the matching retrospective baseline variable. We assessed collinearity between selected variables before final model construction. Results are presented following STROBE guidelines; odds ratios (ORs), proportional odds, or β coefficients with 95% CIs are reported for comparator groups, as appropriate, based on model form.

We used complete case analysis and conducted all analyses using SAS version 9.4 (SAS Institute Inc., https://www.sas.com). We considered p<0.05 to be statistically significant for all analyses conducted. 

## Results

**Figure 2:**
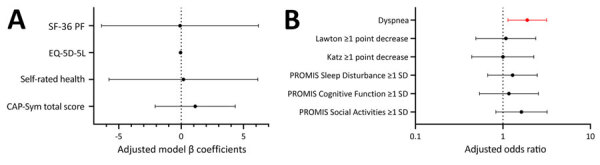
Multivariable model results for 6- to 12-month primary and secondary outcomes for patients in a study of long-term illness in adults hospitalized for respiratory syncytial virus disease or COVID-19, United States, February 2022–September 2023. The earliest completed survey from 6, 9, or 12 months was included. Results are presented separately for continuous (A) and binary or ordinal (dyspnea) (B) outcomes. Models were additionally adjusted for sex, race/ethnicity, smoking status, baseline functional limitations, number of organ systems affected by chronic disease. For outcome models with baseline data available (Katz, Lawton, and SF-36 PF), the matching retrospective baseline variable was included. Red indicates statistically significant effects. Error bars indicate 95% CIs. Vertical dotted lines indicate a null result value for that model type. The following are those outcomes where higher values indicate worse illness for those with respiratory syncytial virus: CAP-Sym total score, dyspnea, Lawton >1 point decrease, Katz >1 point decrease, and PROMIS Sleep Disturbance >1 SD. Details on each testing scale are provided in the text.

During February 2022–September 2023, a total of 21,611 patients were enrolled in IVY during acute hospitalization. Of those, 610 were hospitalized with RSV, and 465 were eligible and approached for long-term outcome assessment (Appendix 2 Figure 2). Sixty-two patients had co-detection of influenza or COVID-19 and were excluded from the analysis. Of the remaining 403 patients, 146 completed surveys included in the analysis at the earliest of 6 (n = 84), 9 (n = 34), or 12 (n = 28) months. Patients hospitalized with RSV participating in SunRISE were largely similar to nonparticipants, although nonparticipants were older and more often discharged to LTCFs (Appendix 2 Table 2). An additional 118 patients hospitalized with COVID-19 (of 8,715 hospitalized) were able to be matched to RSV cases and included as a comparator cohort. The median time of survey follow-up was ≈6.5 (IQR 5.2–9.8) months for those hospitalized with RSV and 6.7 (IQR 5.6–9) months for those hospitalized with COVID-19.

### Characteristics of RSV-Positive Participants

Median age of participating RSV patients was 60.5 (IQR 49.0–70.0) years; 88 (60.3%) were female and 58 (39.7%) male, 43 (29.5%) were non-Hispanic Black, and 22 (15.1%) were of Hispanic ethnicity (Table 1). Most (67.8%) patients had cardiovascular disease before hospitalization with RSV; other common underlying conditions included pulmonary disease (45.9%), endocrine disease (36.3%), and immunocompromised status (30.1%). Forty-four (30.1%) patients had baseline physical functioning limitations. During hospitalization with RSV, 25% were admitted to an ICU, and 36% had severe in-hospital outcomes; median hospital length of stay was 5 (IQR 3–9) days.

**Table 1:** Characteristics of patients in a study of long-term illness in adults hospitalized for respiratory syncytial virus disease or COVID-19, United States, February 2022–September 2023*

Category	Primary RSV cohort			COVID-19, n = 118
	Overall, n = 146	Age <60, n = 71	Age >60, n = 75	
Demographics				
Age at admission, y (IQR)	60.5 (49.0–70.0)	49.0 (35.0–54.0)	70.0 (65.0–75.0)	64.5 (52.0–75.0)
Sex				
F	88 (60.3)	39 (54.9)	49 (65.3)	60 (50.9)
M	58 (39.7)	32 (45.1)	26 (34.7)	57 (48.3)
Race/ethnicity				
Non-Hispanic White	68 (46.6)	22 (31.0)	46 (61.3)	73 (61.9)
Non-Hispanic Black	43 (29.5)	26 (36.6)	17 (22.7)	27 (22.9)
Hispanic	22 (15.1)	15 (21.1)	7 (9.3)	11 (9.3)
Other	8 (5.5)	4 (5.6)	4 (5.3)	5 (4.2)
Unknown	5 (3.4)	4 (5.6)	1 (1.3)	2 (1.7)
Current/former smoker	22 (15.1)	12 (16.9)	10 (13.3)	21 (18.8)
Long-term care facility at admission†	4 (2.7)	1 (1.4)	3 (4.0)	5 (4.2)
Baseline characteristics at hospital admission				
Immunocompromised status	44 (30.1)	28 (39.4)	16 (21.3)	39 (33.1)
Cardiovascular disease	99 (67.8)	40 (56.3)	59 (78.7)	82 (69.5)
Neurologic disease	9 (6.2)	8 (11.3)	1 (1.3)	19 (16.1)
Pulmonary disease	67 (45.9)	25 (35.2)	42 (56.0)	38 (32.2)
Gastrointestinal disease	6 (4.1)	6 (8.5)	0 (0.0)	7 (5.9)
Endocrine disease	53 (36.3)	23 (32.4)	30 (40.0)	51 (43.2)
Renal disease	40 (27.4)	15 (21.1)	25 (33.3)	34 (28.8)
Hematologic disease	24 (16.4)	9 (12.7)	15 (20.0)	16 (13.6)
Autoimmune/inflammatory disease	13 (8.9)	8 (11.3)	5 (6.7)	9 (7.6)
Psychiatric disorders	32 (21.9)	18 (25.4)	14 (18.7)	32 (27.1)
No. organ systems with chronic disease (IQR)‡	2.0 (2.0–3.0)	2.0 (1.0–3.0)	2.0 (2.0–3.0)	2.0 (1.0–3.0)
Baseline physical functioning limitations§	44 (30.1)	18 (25.4)	26 (34.7)	50 (42.4)
COVID-19 vaccination, current season¶	50 (34.2)	16 (22.5)	34 (45.3)	36 (30.5)
Characteristics of hospital course				
Intensive care unit admission	36 (24.7)	19 (26.8)	17 (22.7)	15 (12.7)
Severe hospital outcomes#	53 (36.3)	24 (33.8)	29 (38.7)	20 (17.0)
Hospital length of stay, d (IQR)**	5.0 (3.0–9.0)	4.0 (3.0–8.0)	5.0 (3.0–10.0)	4.0 (2.0–7.0)
Discharged to long-term care facility	10 (6.9)	4 (5.6)	6 (8.0)	15 (12.7)

*Values are no. (%) except as indicated. Percentages for fields with missing values are computed based on the full column value. For those with RSV, variables missing data were current/former smoker (n = 12), long-term care facility at admission (n = 2), COVID-19 vaccination status (n = 2), and discharged to long-term care facility (n = 6). For those with COVID-19, variables missing data were sex (n = 1), current/former smoker (n = 6), long-term care facility at admission (n = 4), COVID-19 vaccination status (n = 4), and discharged to long-term care facility (n = 1). IQR, interquartile range; RSV, respiratory syncytial virus. †Including nursing homes, assisted living homes, and rehabilitation hospital or other subacute or chronic facility. ‡Organ systems affected by chronic disease: cardiovascular disease, neurologic disease, pulmonary disease, gastrointestinal disease, endocrine disease, kidney disease, hematologic disease, autoimmune disease, and immunocompromising conditions. §Baseline physical functioning limitations are defined as receiving home care help or unable to walk. ¶Defined for those hospitalized before September 1, 2022, as completion of a primary series plus 1 or 2 monovalent (original) boosters and for those hospitalized after September 1, 2022, as receipt of >1 bivalent vaccine doses #Defined as any of the following events during the acute illness hospitalization: deep vein thrombosis, pulmonary embolism, myocardial infarction, stroke, high-flow nasal cannula oxygen, noninvasive or invasive mechanical ventilation, new tracheostomy, new renal replacement therapy, use of vasopressors, or extracorporeal membrane oxygenation. **Those hospitalized >28 d had length of stay truncated at 28 d.

### Long-Term Outcomes after RSV Hospitalization

Compared with preillness baseline, 28% of patients had a decrease of >5 points in their SF-36 Physical Function Subscale scores 6–12 months after hospitalization, indicating a significant loss of physical function after acute RSV illness (Table 2; Appendix 2 Table 3); 48% reported similar physical functioning, and 12% reported improvement. In addition, 11.6% had a loss in instrumental and 11.0% in basic ADL; 71% (instrumental) and 76% (basic) reported the same ADLs as baseline, and ≈10% showed improvement. More than 25% reported that they got breathless when dressing, talking, or at rest. The RSV cohort had a median (IQR) self-rated health of 60 (IQR 50–80), indicating moderate health. Last, 21.9% indicated that they had poor quality of life as characterized by the EQ-5D-5L.

**Table 2:** Six- to 12-mo outcomes in a study of long-term illness in adults hospitalized for respiratory syncytial virus disease or COVID-19, United States, February 2022–September 2023*

Category	RSV only					RSV versus COVID-19		
	Age <60, n = 71	Age >60, n = 75		p value†		RSV positive, n = 146	SARS-CoV-2 positive, n = 118	p value†
Primary outcomes‡								
SF36-PF, median (IQR)	47.5 (20.0–80.0)	20.0 (10.0–60.0)		0.008		40.0 (15.0–75.0)	42.5 (10.0–80.0)	0.799
Change from baseline, median (IQR)	0.0 (–5.0 to 0.0)	0.0 (–10.0 to 0.0)		0.087		0.0 (–5.0 to 0.0)	0.0 (–10.0 to 0.0)	0.462
Katz ADLs,§ median (IQR)	6.0 (6.0–6.0)	6.0 (5.0–6.0)		0.053		6.0 (5.0–8.0)	6.0 (5.0–6.0)	0.854
Decrease from baseline >1 point	7 (9.9)	9 (12.0)		0.682		16 (11.0)	12 (10.2)	0.858
Lawton instrumental ADLs,§ median (IQR)	8.0 (6.0–8.0)	7.0 (3.0–8.0)		0.077		8.0 (5.0–8.0)	8.0 (5.0–8.0)	0.802
Decrease from baseline >1 point	3 (4.2)	14 (18.7)		0.005		17 (11.6)	17 (14.4)	0.519
Dyspnea				0.558				0.104
Grade 0/1	21 (29.6)	22 (29.3)				43 (29.5)	49 (41.5)	
Grade 2	9 (12.7)	6 (8.0)				15 (10.3)	7 (5.9)	
Grade 3	19 (26.8)	18 (24.0)				37 (25.3)	26 (22.0)	
Grade 4	16 (22.5)	24 (32.0)				40 (27.4)	23 (19.5)	
Self-rated health, median (IQR)	62.5 (50.0–80.0)	60.0 (50.0–80.0)		0.222		60.0 (50.0–80.0)	70.0 (50.0–80.0)	0.678
EQ-5D-5L, median (IQR)	0.712 (0.394–0.926)	0.687 (0.363–0.883)		0.551		0.705 (0.338–0.902)	0.719 (0.458–0.904)	0.481
Good, >0.632	39 (54.9)	39 (52.0)				78 (53.4)	67 (56.8)	
Fair, 0.338–0.632	13 (18.3)	15 (20.0)				28 (19.2)	24 (20.3)	
Poor, <0.338	16 (22.5)	16 (21.3)				32 (21.9)	22 (18.6)	
Secondary outcomes								
PROMIS Sleep Disturbances								
Median (IQR)	53.9 (41.2–63.8)	51.4 (41.2–57.2)		0.219		51.7 (41.2–61.1)	50.0 (42.1–57.7)	0.606
>1 SD >50	25 (35.2)	14 (18.7)		0.023		39 (26.7)	23 (19.5)	0.189
PROMIS Cognitive Function								
Median (IQR)	50.8 (43.4–66.2)	52.7 (43.4–66.2)		0.931		51.4 (43.4–66.2)	51.7 (43.9–66.2)	0.880
>1 SD <50	8 (11.3)	12 (16.0)		0.333		20 (13.7)	16 (13.6)	0.963
PROMIS Social Activities								
Median (IQR)	51.8 (37.2–64.2)	49.9 (40.2–58.1)		0.489		51.5 (37.9–64.2)	51.8 (40.3–64.2)	0.423
>1 SD <50	19 (26.8)	16 (21.3)		0.626		35 (24.0)	26 (22.0)	0.666
CAP-Sym Score								
Total score	14.0 (3.0–24.0)	12.0 (6.0–20.0)		0.533		13.0 (5.0–23.0)	9.0 (3.0–20.0)	0.161
Total no. severe symptoms	1.0 (0.0–3.0)	1.0 (0.0–2.0)		0.269		1.0 (0.0–3.0)	1.0 (0.0–2.0)	0.430
Exploratory outcomes§								
Receives regular help at home with medical care or ADL	27 (38.0)	38 (50.7)		0.125		65 (44.5)	53 (44.9)	0.900
New receipt of home health care from hospitalization	10 (14.1)	17 (22.7)		0.195		27 (18.5)	10 (8.5)	0.019
SNF/LTCF at survey timepoint	2 (2.8)	7 (9.3)		0.094		9 (6.2)	7 (5.9)	0.784
New SNF/LTCF compared with hospitalization	1 (1.4)	5 (6.7)		0.099		6 (4.1)	2 (1.7)	0.263
Patient missed work or school¶	12/18 (66.7)	4/9 (44.4)		0.411		16/27 (59.3)	11/19 (57.9)	0.926
Caregiver missed work or school	31 (43.7)	18 (24.0)		0.016		49 (34.5)	29 (24.6)	0.196
New/worsened home oxygen use#	19 (26.9)	17 (22.7)		0.566		36 (24.7)	19 (16.1)	0.110
New/worsened CPAP/other breathing machine use#	6 (8.5)	5 (6.7)		0.683		11 (7.5)	5 (4.2)	0.287

*Values are no. (%) except as indicated. Earliest completed survey from 6, 9, or 12 mo was used. Percentages for fields with missing values are computed based on the full column. Details on each testing scale are provided in the text. For those with RSV, variables missing data were SF36-PF (n = 9), baseline SF-36 PF comparison (n = 17), Katz (n = 5), baseline Katz comparison (n = 6), Lawton (n = 4), baseline Lawton comparison (n = 11), dyspnea (n = 11), self-rated health (n = 9), EQ-5D-5L (n = 8), PROMIS sleep disturbance (n = 4), PROMIS cognitive function (n = 8), PROMIS social activities (n = 14), new home care help (n = 3), new LTCF (n = 3), and caregiver time off (n = 4). For those with COVID-19, variables missing data were SF36-PF (n = 8), baseline SF-36 PF comparison (n = 12), Katz (n = 5), baseline Katz comparison (n = 6), Lawton (n = 5), baseline Lawton comparison (n = 8), dyspnea (n = 13), self-rated health (n = 4), EQ-5D-5L (n = 5), PROMIS sleep disturbance (n = 5), PROMIS cognitive function (n = 6), PROMIS social activities (n = 10), home care help (n = 1), new home care help (n = 2), new LTCF (n = 4), caregiver time off (n = 10), new home oxygen use (n = 3), and new home CPAP use (n = 3). Of those with RSV, 94 (64%) had nonmissing data for the survey components, and of those with COVID-19, 76 (64%) had nonmissing data for the survey components; missing data was imputed to 0/no for symptoms, so those missing individual symptoms are not included. ADL, activity of daily living; CPAP, continuous positive airway pressure; EQ-5D-5L, EuroQol 5-dimension, 5-level questionnaire; IQR, interquartile range; LTCF, long-term care facility; RSV, respiratory syncytial virus; SF-36 PF, Short Form-36 Physical Function Subscale score; SNF, skilled nursing facility. †p values compare persons <60 y of age to those >60 y of age and patients with RSV versus those with COVID-19 and were computed using χ^2^ or Fisher exact tests for categorical variables or Wilcoxon signed-rank tests for continuous variables, as appropriate. ‡Change from baseline calculated for those surveys with nonmissing data. §Basic ADLs include bathing, feeding, and dressing; instrumental ADLs include shopping, managing finances, or making telephone calls ¶Column percentages computed for those who reported being employed or in school at time of hospitalization. ¶Compared with 1 mo before hospitalization.

Among the RSV cohort, 26.7% of participants reported PROMIS Sleep Disturbances >1 SD above the standardized mean of 50, 13.7% reported PROMIS Cognitive scores >1 SD below the standardized mean of 50, and 24.0% reported PROMIS Social Activities >1 SD below the standardized mean of 50. The total median modified CAP-Sym score was 13 (IQR 5–23), with a median of 1 (IQR 0–3) severe symptom reported (Appendix 2 Table 4).

Almost half (44%) of RSV participants reported receiving home care help for medical care or activities of daily living at the time of the survey. Of those currently working (n = 27), 59% reported missing >1 day of work or school because of illness after hospitalization.

### Comparing 6–12-Month Outcomes for Patients <60 and >60 Years of Age in Adults Hospitalized for RSV

The loss of ability to perform >1 basic ADL, decreased physical function (SF-36 Physical Function Subscale decrease >5 points), extreme dyspnea, self-rated health, and poor quality of life were similar between younger (<60 years) and older (>60 years) patients with RSV at 6–12 months postillness. Cognitive function, social function, and CAP-Sym scores were additionally similar. However, younger adults had lower odds of a loss in ability to perform 1 instrumental ADL (4.2% vs. 18.7%; adjusted OR [aOR] 0.24, 95% CI 0.07–0.83) (Figure 1) and higher odds of having sleep disturbances >1 SD above the mean (35.2% vs 18.7%; aOR 2.61, 95% CI 1.11–6.12) compared with older patients.

### Comparing 6–12-Month Outcomes for Adults Hospitalized with RSV Versus COVID-19

Compared with COVID-19 patients and similar to the larger IVY cohort (*4*), patients hospitalized with RSV in SunRISE were slightly younger (median age 60.5 [IQR 49.0–70.0] years vs. 64.5 [IQR 52.0–75.0] years) and had higher proportions of preillness pulmonary disease (45.9% vs. 32.2%). Those with RSV additionally had more frequent ICU admission (24.7% vs. 12.7%), and severe in-hospital outcomes (36.3% vs. 17.0%) than those with COVID-19. Patients hospitalized with COVID-19 reported a higher rate of baseline physical functioning limitations (42.4% vs. 30.1%). Unadjusted outcomes were largely similar for patients with COVID-19 and those with RSV (Table 2). After adjusting for demographics and covariates, those hospitalized with RSV had 1.90 (95% CI 1.14–3.16) times higher proportional odds of more severe dyspnea than those hospitalized with COVID-19 (Figure 2; Appendix 2 Table 5); results were similar when adjusting for pulmonary disease. We found no other statistically significant differences between outcomes.

## Discussion

In this multicenter analysis of adults hospitalized with RSV in 20 US states, patients exhibited significant impairments in physical function and abilities to perform ADLs, significant dyspnea, poor self-rated health, and poor quality of life at 6–12 months after hospitalization. Of note, almost half of SunRISE participants were <60 years of age, the minimum age recommended for RSV vaccination by the Advisory Committee on Immunization Practices starting in June 2023 (*16*). However, our cohort showed significant and persistent functional and quality of life impacts after RSV hospitalization regardless of age. In addition, those hospitalized with RSV suffered a constellation of postdischarge sequelae of similar breadth and severity to that described by patients hospitalized with COVID-19, with the exception of more severe dyspnea in those with RSV. Together, those results suggest the potential for substantial and lingering harm from severe RSV illness across the entire adult age spectrum. 

Our findings document long-term sequelae after hospitalization with RSV. One potential additional benefit of RSV vaccination may be prevention of adverse long-term outcomes. This information can be used by clinicians and public health practitioners who monitor at-risk patients after hospitalization, and to inform efforts to prevent and reduce the severity of these hospitalizations through targeted vaccination campaigns and other measures (*34*).

Few studies have evaluated the effect of severe RSV illness on physical function, which can deteriorate even before any impairments in performing activities of daily living are appreciated. We found that the median SF-36 Physical Function Subscale score at the time of the 6–12 month follow-up survey was 40, and >25% of all participants reported a decrease of >5 points (>10 points for those >60 years of age) from baseline, indicating substantial physical long-term impairment after hospitalization with RSV. That loss in physical function could be driven by persistent symptoms; 63% of patients reported moderate or worse dyspnea at the time of the survey. We also observed that 11% of our cohort had lost the ability to perform 1 basic ADL and 12% lost the ability to perform 1 instrumental ADL at 6-months. Those percentages are lower than those observed in a previous study that reported 33% lost the ability to perform 1 basic ADL and 32% lost the ability to perform 1 instrument ADL at 6 months after RSV hospitalization (*15*). However, those differences are likely because of different patient characteristics; our cohort was younger (median age 60.5 vs. 74 years), and fewer resided in a skilled nursing facility (3% vs. 8%).

Given the degree of functional impairment, disability, and persistent symptoms, that SunRISE participants reported poor self-rated health and quality of life is not surprising. Several studies have evaluated health-related quality of life after RSV illness. A study in Europe observed a median (IQR) EQ-5D-5L index score of 0.85 (0.81–0.94) in community-dwelling older patients with RSV illness at 1 week after acute illness, but index scores improved to baseline 4 weeks postinfection (*35*). However, that cohort enrolled nonhospitalized RSV patients, who likely had better baseline quality of life and health than those in our study; health-related quality of life and self-rated health are moderately correlated with lower respiratory tract symptoms (*14*). Another study collected EQ-5D-5L scores at 3 months after hospitalization with RSV from 238 patients; the authors did not report summary EQ-5D-5L index scores, but the mean self-rated health was slightly higher than that observed in SunRISE (*14*). Our study extends the findings to a younger cohort with longer duration of follow-up and strengthens evidence for potential significant long-term sequelae after RSV hospitalization, regardless of age. Additional studies are needed to determine how severe RSV illness affects quality of life.

This study has several strengths, most notably that the underlying patient population is drawn from a large nationwide public health surveillance network for ARI, making the cohort more generalizable to those experiencing severe RSV illness. Robust data collection, including surveys in both English and Spanish, increase the generalizability of the SunRISE cohort. Surveys included both single-item questions and validated questionnaires, enabling robust data capture and comparison to other critical care and ARI cohorts. 

The first limitation of our study is that, as for many prospective studies, the included population may represent a relatively healthier population; survival to hospital discharge was required for participation, so results may underestimate RSV and COVID-19 posthospitalization sequelae. In addition, given the broad range of underlying conditions in this population, fully separating changes occurring as part of the natural history of those conditions versus the effects of hospitalization for acute viral illness may be difficult. That difference could potentially overestimate the degree of long-term sequelae caused by the acute viral disease. However, adjustment for functional limitations collected at hospitalization, number of organ systems affected by a chronic disease, and pulmonary disease status (for dyspnea) reduced that risk. Retrospective recall of preillness health status is imprecise because of time elapsed and other factors, and is potentially biased in either direction. Further, prehospitalization baseline data were limited and not collected on all outcomes, potentially biasing results in either direction. Finally, the sample size for this study did not allow for the robust examination of risk factors or protective factors, such as vaccination, for long-term sequelae after hospitalization with RSV; those considerations will be the focus of subsequent analyses of the SunRISE program.

In this first analysis of the SunRISE program, many patients who were hospitalized with RSV had poor physical functioning (median SF-36 Physical Function Subscale score of 40 out of 100), functional impairment (19% reporting new receipt of home help after hospitalization), and persistent symptoms including dyspnea (63% reporting grade 2 or higher dyspnea) at 6–12 months after hospitalization, regardless of age. A substantial proportion of participants also reported poor quality of life (41% reporting fair or poor EQ-5D-5L scores) and poor self-rated health (median 60 out of 100) up to 1 year after hospitalization with RSV. Such long-term effects appear similar to those occurring after hospitalization with COVID-19, another acute respiratory illness associated with adverse long-term outcomes in adults. Data from this analysis can inform risk communication about RSV in adults and the potential benefits of RSV prevention through vaccination.

Appendix 1 Collaborators in the IVY Network for long-term illness in adults hospitalized for respiratory syncytial virus disease, United States, February 2022–September 2023.

Appendix 2 Additional information for long-term illness in adults hospitalized for respiratory syncytial virus disease, United States, February 2022–September 2023.
