# Supplementary material for: Long-Term Illness in Adults Hospitalized for Respiratory Syncytial Virus Disease, United States, February 2022–September 2023
Source: Emerg Infect Dis. 2025 Dec;31(Suppl 2):S20–9. doi: 10.3201/eid3114.241982 (PMC12829483; doi:10.3201/eid3114.241982)
Supplement: Appendix 1 — Collaborators in the IVY Network for long-term illness in adults hospitalized for respiratory syncytial virus disease, United States, February 2022–September 2023. [file 24-1982-Techapp-s1.pdf]

Article DOI: https://doi.org/10.3201/eid3114.241982

EID cannot ensure accessibility for supplementary materials supplied by authors. Readers who have difficulty accessing supplementary content should contact the authors for assistance.

Long-Term Illness in Adults Hospitalized for Respiratory Syncytial Virus Disease, United States, February 2022–September 2023

Appendix 1

Investigators and Collaborators in the Investigating Respiratory Viruses in the Acutely Ill (IVY) Network

Baylor, Scott and White, Temple and Dallas, Texas

Manju Gaglani, Shekhar Ghamande, Tresa McNeal, Cristie Columbus, Robert L. Gottlieb, Catherine Raver, Ashley Bychkowsky, Symone Dunkley, Tammy Fisher, Daniela Gonzalez, Therissa Grefsrud, Mariana Hurutado-Rodriguez, Gabriela Perez

Baylor University Medical Center, Dallas, Texas

Ashley Bychkowski, Symone Dunkley, Tammy Fisher, Daniela Gonzalez, Therissa Grefsrud, Mariana Hurutado-Rodriguez, Gabriela Perez

Baystate Medical Center, Springfield, Massachusetts

Jay Steingrub, Lesley De Souza, Scott Ouellette, Cynthia Kardos, Rae Lynn Defeo

Beth Israel Medical Center, Boston Massachusetts

Nathan I. Shapiro, Michael Bolstad, Brianna Coviello, Robert Ciottone, Arnaldo Devilla, Ana Grafals, Conor Higgins, Carlo Ottanelli, Kimberly Redman, Douglas Scaffidi, Alexander Weingart

Centers for Disease Control and Prevention (CDC), Atlanta, Georgia

Diya Surie, Fatimah S. Dawood, Sharon Saydah, Sascha Ellington, Nathaniel M. Lewis

Cleveland Clinic, Cleveland, Ohio

Omar Mehkri, Megan Mitchell, Zachary Griffith, Connery Brennan, Kiran Ashok, Bryan Poynter, Abhijit Duggal

Emory University, Atlanta, Georgia

Laurence Busse, William Bender, Caitlin ten Lohuis

Hennepin County Medical Center, Minneapolis, Minnesota

Matthew Prekker, Audrey Hendrickson, Mary O'Rourke, Leyla Taghizadeh, Anne Frosch

Henry Ford Health, Detroit, Michigan

Ivana A Vaughn, Mayur Ramesh, Lois E Lamerato, Kim Beney, Jean Ashley Lava, Melissa Resk, Sindhuja Koneru, Rachna Jayaprakash, Zina Pinderi

Intermountain Medical Center, Murray, Utah

Ithan D. Peltan, Samuel M. Brown, Joslyn Bassett, Shandi Poulson, Vineela Thumma

Johns Hopkins University, Baltimore, Maryland

David N. Hager, Harith Ali, Safa Saeed

Montefiore Medical Center, Bronx, New York

Michelle Gong, Amira Mohamed

Ohio State Medical Center, Columbus, Ohio

Matthew Exline, Madison So, Jun Sung Park, Manisha Pathak, David Smith, Rasha Alrifae, Gabrielle Swoope, Maryiam Khan, Amanie Rasul, Brooke Lee, Zachery Lewald, Reece Wilson, Connor Snyder, Sarah Karow

Oregon Health and Sciences University, Portland, Oregon

Akram Khan, Adrian Hernandez-Frausto, Edvinas Pocius, Emily Tribbett, Genesis Briceno, Jose Pena, Gopal Allada, Tomas Cordova, Bethany Collins, Sherie Gause, Kinsley Hubel, Jonathan Pak

Stanford University, Stanford, California

Jennifer G. Wilson, Cynthia Perez, Grace Kyin-Ye Tam, Vanessa Pitre, Lily Lau, Leonard Basobas, Alexandra June Gordon, Samantha Ferguson

University of Arizona, Tucson, Arizona

Karen Lutrick, Cameron Hypes, Elizabeth Salvagio Campbell, Mary Labus, Bailey Bowles, Frances Nagore

University of California-Los Angeles, Los Angeles, California

Nida Qadir, Steven Y. Chang, Cody Tran, Sukantha Chandrasekaran, Omai Garner

University of Colorado, Aurora, Colorado

Adit A. Ginde, Samantha Simon, Amanda Martinez, Amy Sullivan, Laura Aguilar-Marquez, Erika Alor, Yvette Evans, Jacob Rademacher

University of Iowa, Iowa City, Iowa

Nicholas Mohr, Anne Zepeski, Paul Nassar, Noble Briggs, Jacob Hampton, Cathy Fairfield

University of Miami, Miami, Florida

Chris Mallow, Carolina Rivas

University of Michigan, Ann Arbor, Michigan

Emily Martin, Adam Lauring, Aleda M. Leis, Weronika Valvano, Arnold S. Monto

University of Utah, Salt Lake City, Utah

Estelle S. Harris, Rylie McBride, Bryce Bosworth

University of Washington, Seattle, Washington

Nicholas Johnson, Joshua Acidera, Maile McKnown, Dylan Clark, Amol Gajendragadkar

Vanderbilt University Medical Center, Nashville, Tennessee

Wesley H. Self, Yuwei Zhu, Kelsey N. Womack, Jillian P. Rhoads, Adrienne Baughman, Sydney A. Swan, Cassandra A. Johnson, Colleen Ratcliff, Jakea Johnson, Karen F. Miller

Wake Forest University, Winston-Salem, North Carolina

Kevin Gibbs, Hannah Strait, Darija Ward, Lisa Parks, Leigha Landreth

Washington University, St. Louis, Missouri

Jennie Kwon, Bijal Parikh, David McDonald, Carleigh Samuels, Lucy Vogt, Caroline O'Neil, Alyssa Valencia, Francesca Yerbic, Olivia Arter, Kim Vu

Yale University, New Haven, Connecticut

Basmah Safdar, Anirudh Goyal, Ivan Velasquez, Lauren DeLamielleure, Uchechi Okoronkwo, Carolyn Brokowski
