# Supplementary material for: Long-Term Illness in Adults Hospitalized for Respiratory Syncytial Virus Disease, United States, February 2022–September 2023
Source: Emerg Infect Dis. 2025 Dec;31(Suppl 2):S20–9. doi: 10.3201/eid3114.241982 (PMC12829483; doi:10.3201/eid3114.241982)
Supplement: Appendix 2 — Additional information for long-term illness in adults hospitalized for respiratory syncytial virus disease, United States, February 2022–September 2023. [file 24-1982-Techapp-s2.pdf]

Article DOI: https://doi.org/10.3201/eid3114.241982

EID cannot ensure accessibility for supplementary materials supplied by authors. Readers who have difficulty accessing supplementary content should contact the authors for assistance.

Long-Term Illness in Adults Hospitalized for Respiratory Syncytial Virus Disease, United States, February 2022–September 2023

Appendix 2

Appendix 2 Table 1. SunRISE survey domains and outcome measures

Category	Assessment type	Domain	Description
Independence	Residence	Residence	Single item
	Home help	Home help	Single item
	Katz	Basic Activities of Daily Living	5 items (bathing, dressing, toileting, transferring, and feeding)
	Lawton	Instrumental ADLs	8 items (using telephone, shopping, preparing food, housekeeping, laundry, transportation, medications and finances)
Symptoms	Community-acquired pneumonia symptoms survey (modified)	Symptoms	Presence and impact of respiratory and systemic symptoms (15 items)
	MRC Dyspnea Scale (modified)	Shortness of breath	Single item (5 levels)
	PROMIS-Sleep Disturbance SF 4a	Sleep	4 items on severity and characteristics of sleep
Function	PROMIS Cognitive Abilities v.2.0 - Short Form 6	Global Cognition	6 items of perception of attention, concentration, memory, processing speed, and cognitive fatigue
	SF-36-physical functioning subscale	Physical function	10-item questionnaire focused on physical functional limitations
QOL/ Recovery	EQ-5D-5L	Quality of Life	6 items evaluating quality of life: mobility, self-care, usual activities, pain, anxiety/depression, and overall
	Self-rated health	Quality of Life	Single item asking participant to rate health on a scale from 0-100
Impact	Work/school missed (patient)	Productivity	Single item that asks if patient missed work or school due to health concerns over preceding period
	Work/school missed (caregiver)	Productivity	Single item that asks if caregiver missed work or school due to health concerns over preceding period
	PROMIS- Social Roles and Activities SF-4a	Participation	Four questions about social roles and activities
Health care	Respiratory support	Respiratory support	One question asking about new or increased need for supplemental O2 and/or positive pressure ventilation

Appendix 2 Table 2. Characteristics of those hospitalized with RSV participating in SunRISE and included in the current analysis compared to those hospitalized with RSV not included in the current analysis*

Category	RSV+ Participating in SunRISE and included in analysis (N = 146)	All other RSV+ (N = 464)	P-Value
Demographics			
Age at admission	60.5 [49.0–70.0]	66.0 [53.0–76.5]	<0.001
Female sex	89 (61.0)	245 (52.8)	0.084
Race/Ethnicity			0.292
Non-Hispanic White	68 (46.6)	245 (52.8)	
Non-Hispanic Black	43 (29.5)	100 (21.6)	
Hispanic	22 (15.1)	81 (17.5)	
Other	8 (5.5)	28 (6.0)	
Unknown	5 (3.4)	10 (2.2)	
Long term care facility at admission†	6 (4.1)	41 (8.8)	0.110
Baseline Characteristics at Hospitalization			
Immunocompromised Status	44 (30.1)	134 (28.9)	0.771
Cardiovascular Disease	99 (67.8)	334 (72.0)	0.332
Neurological Disease	9 (6.2)	73 (15.7)	0.003
Pulmonary Disease	67 (45.9)	201 (43.3)	0.585
Gastrointestinal Disease	6 (4.1)	30 (6.5)	0.292
Endocrine Disease	53 (36.3)	183 (39.4)	0.497
Renal Disease	40 (27.4)	110 (23.7)	0.367
Hematological Disease	24 (16.4)	72 (15.5)	0.790
Autoimmune/Inflammatory Disease	13 (8.9)	30 (6.5)	0.315
Psychiatric Disorders	32 (21.9)	108 (23.3)	0.734
Number of organ systems with chronic disease‡	2.0 [2.0-3.0]	2.0 [2.0-3.0]	0.493
COVID-19 vaccination, current season§	50 (34.7)	133 (29.3)	0.218
Hospitalization Characteristics			
Intensive care unit admission	36 (24.7)	88 (24.4)	0.960
Severe hospital outcomes¶	53 (36.3)	139 (30.0)	0.150
In-hospital mortality#	0 (0.0)	23 (5.0)	NA
Hospital length of stay (days)**	5.0 [3.0-9.0]	5.0 [3.0-8.0]	0.687
Discharged to long term care facility	10 (6.9)	53 (11.4)	<0.001

*Columns are mutually exclusive. Data are presented as median [interquartile range] or frequency (column percent), as applicable. Percentages for fields with missing values are computed based on the full column n. Note data presented here are from original IVY enrollment; slight discrepancies exist with variables confirmed at SunRISE initial contact for those participating in SunRISE (sex, home type).

†Long term care facilities include: nursing homes, assisted living homes, rehabilitation hospital/other subacute or chronic facility

‡Organ systems affected by chronic disease include: cardiovascular disease, neurologic disease, pulmonary disease, gastrointestinal disease, endocrine disease, kidney disease, hematologic disease, autoimmune disease, and immunocompromising conditions

§Defined for those hospitalized before September 1, 2022 as completion of a primary series plus 1 or 2 monovalent (original) boosters and for those hospitalized after September 1, 2022 as receipt of one or more bivalent vaccine doses

¶Defined as any of the following events during the acute illness hospitalization: deep vein thrombosis, pulmonary embolism, myocardial infarction, stroke, high-flow nasal cannula oxygen, non-invasive or invasive mechanical ventilation, new tracheostomy, new renal replacement therapy, or use of vasopressors, extracorporeal membrane oxygenation.

#In-hospital mortality within 28 days of hospital admission (missing n=101)

**Those hospitalized beyond 28 days have length of stay truncated at 28 days

Appendix 2 Table 3. Summary statistics for surveys by first timepoint six months or later with data available for those hospitalized with RSV*

Outcomes	6 Month Survey (N = 84)	9 Month Survey (N = 34)	12 Month Survey (N = 28)
Primary			
SF36 - PF	40.0 [10.0–70.0]	32.5 [10.0–70.0]	50.0 [15.0–85.0]
Change from baseline†	0.0 [-10.0 to 0.0]	0.0 [-5.0–0.0]	0.0 [0.0–0.0]
Katz ADLs	6.0 [5.0–6.0]	6.0 [5.0–6.0]	6.0 [4.0–6.0]
Decrease from baseline ≥1 point†	7 (8.3)	7 (20.6)	2 (7.1)
Lawton IADLs	8.0 [5.0-8.0]	7.0 [5.0–8.0]	8.0 [4.0–8.0]
Decrease from baseline ≥1 point†	12 (14.3)	3 (8.8)	2 (7.1)
Dyspnea			
Grade 0/1	25 (29.8)	9 (26.5)	9 (32.1)
Grade 2	12 (14.3)	2 (5.9)	1 (3.6)
Grade 3	17 (20.2)	9 (26.5)	11 (39.3)
Grade 4	25 (29.8)	11 (32.4)	4 (14.3)
Self-rated health	60.0 [50.0–80.0]	60.0 [50.0–80.0]	70.0 [50.0–97.0]
EQ-5D-5L	0.716 [0.429–0.904]	0.543 [0.295-0.836]	0.695 [0.445–0.904]
Good (>0.632)	47 (56.0)	16 (47.1)	15 (53.6)
Fair (0.338 – 0.632)	14 (16.7)	8 (23.5)	6 (21.4)
Poor (<0.338)	17 (20.2)	10 (29.4)	5 (17.9)
Secondary			
PROMIS Sleep Disturbances			
Median (IQR)	52.8 [41.9–60.5]	51.1 [41.9-62.0]	46.4 [38.4–61.1]
≥1SD above 50	21 (25.0)	11 (32.4)	7 (25.0)
PROMIS Cognitive Function			
Median (IQR)	52.7 [43.7–66.2]	53.6 [42.8-66.2]	49.7 [43.4–66.2]
≥1SD below 50	10 (11.9)	4 (11.8)	6 (21.4)
PROMIS Social Activities			
Median (IQR)	51.7 [40.2-58.3]	46.7 [37.2-58.5]	53.6 [37.9–64.2]
≥1SD below 50	19 (22.6)	8 (23.5)	8 (28.6)
CAP-Sym Score			
Total Score	13.5 [5.5–19.0]	11.0 [6.0–26.0]	14.0 [2.5–23.5]
Total Number Severe Symptoms	1.0 [0.0–2.0]	1.0 [0.0–3.0]	1.0 [0.0–3.0]
Exploratory			
Receives regular help at home with medical care or activities of daily living	38 (45.2)	16 (47.1)	11 (39.3)
New receipt of home health care from hospitalization	17 (20.2)	8 (23.5)	2 (7.1)
Skilled nursing facility or long-term care facility at survey timepoint	6 (7.1)	0 (0.0)	3 (10.7)
New SNF/LTC compared to hospitalization	3 (3.6)	0 (0.0)	3 (10.7)
Patient missed work or school if working‡	8/15 (53.3)	4/8 (50.0)	4/4 (100.0)
Caregiver missed work or school	24 (28.6)	14 (41.2)	11 (39.3)
New or worsened home O2 use compared to 1 month before hospitalization	20 (23.8)	9 (26.5)	7 (25.0)
New or worsened CPAP/other breathing machine use compared to 1 month before hospitalization	3 (3.6)	4 (11.8)	4 (14.3)

*Data are presented as median [interquartile range] or frequency (column percent), as applicable.
†Change from baseline calculated for those surveys with non-missing data.
‡Column percentages for patient missed work or school are computed for those who reported being currently employed at time of hospitalization

Appendix 2 Table 4. Symptoms present 6-12 months following hospitalization with RSV and reported severity overall and stratified by age*

Category†	Overall, n = 146	Age <60, n = 71	Age ≥60, n = 75	No. missing‡
CAP-Sym Score – Total Number of Symptoms	5.0 [2.0–7.0]	5.0 [1.0–7.0]	5.0 [2.0-7.0]	NA
Coughing				2
N experienced	65 (44.5)	32 (45.1)	33 (44.0)	
N experienced severe	14 (21.5)	8 (25.0)	6 (18.2)	
Severity	3.0 [2.0–3.0]	3.0 [2.0–3.0]	2.0 [2.0–3.0]	
Wheezing				5
N experienced	46 (31.5)	24 (33.8)	22 (29.3)	
N experienced severe	11 (23.9)	6 (25.0)	5 (22.7)	
Severity	3.0 [2.0–3.0]	3.0 [2.0–3.5]	3.0 [2.0–3.0]	
Chest Pains				4
N experienced	21 (14.4)	13 (18.3)	8 (10.7)	
N experienced severe	5 (23.8)	3 (23.1)	2 (25.0)	
Severity	3.0 [2.0–3.0]	3.0 [2.0–3.0]	2.0 [2.0–4.0]	
Shortness of Breath				1
N experienced	78 (53.4)	34 (47.9)	44 (58.7)	
N experienced severe	32 (41.0)	16 (47.1)	16 (36.4)	
Severity	3.0 [2.0–4.0]	3.0 [2.0–4.0]	3.0 [2.0–4.0]	
Heart Racing/Palpitations				4
N experienced	36 (24.7)	20 (28.2)	16 (21.3)	
N experienced severe	12 (33.3)	7 (35.0)	5 (31.3)	
Severity	3.0 [2.0–4.0]	3.0 [2.0–4.0]	2.5 [1.0–4.0]	
Dizziness/Faintness				5
N experienced	46 (31.5)	22 (31.0)	24 (32.0)	
N experienced severe	13 (28.3)	6 (27.3)	7 (29.2)	
Severity	3.0 [2.0–4.0]	3.0 [3.0–4.0]	2.0 [2.0–4.0]	
Sweating				2
N experienced	28 (19.2)	17 (23.9)	11 (14.7)	
N experienced severe	7 (25.0)	6 (35.3)	1 (9.1)	
Severity	3.0 [2.0–3.5]	3.0 [2.0–4.0]	3.0 [2.0–3.0]	
Chills				2
N experienced	19 (13.0)	8 (11.3)	11 (14.7)	
N experienced severe	4 (21.1)	0 (0.0)	4 (36.4)	
Severity	3.0 [2.0–3.0]	2.5 [1.5–3.0]	3.0 [2.0–4.0]	
Headache				2
N experienced	42 (28.8)	22 (31.0)	20 (26.7)	
N experienced severe	11 (26.2)	5 (22.7)	6 (30.0)	
Severity	2.5 [2.0–4.0]	2.5 [2.0–3.0]	2.5 [2.0–4.0]	
Nausea				2
N experienced	33 (22.6)	19 (26.8)	14 (18.7)	
N experienced severe	10 (30.3)	4 (21.1)	6 (42.9)	
Severity	3.0 [2.0–4.0]	3.0 [2.0–3.0]	3.0 [2.0–4.0]	
Muscle Pain				1
N experienced	68 (46.6)	31 (43.7)	37 (49.3)	
N experienced severe	32 (47.1)	18 (58.1)	14 (37.8)	
Severity	3.0 [3.0–4.0]	4.0 [3.0–4.0]	3.0 [3.0–4.0]	
Lack of Appetite				2
N experienced	40 (27.4)	23 (32.4)	17 (22.7)	
N experienced severe	12 (30.0)	10 (43.5)	2 (11.8)	
Severity	3.0 [2.0–4.0]	3.0 [2.0–5.0]	2.0 [2.0–3.0]	
Trouble Concentrating				3
N experienced	43 (29.5)	19 (26.8)	24 (32.0)	
N experienced severe	12 (27.9)	7 (36.8)	5 (20.8)	
Severity	3.0 [2.0–4.0]	3.0 [3.0–4.0]	2.0 [2.0–3.0]	
Trouble Sleeping				1
N experienced	64 (43.8)	33 (46.5)	31 (41.3)	
N experienced severe	39 (60.9)	24 (72.7)	15 (48.4)	
Severity	4.0 [3.0–5.0]	4.0 [3.0-5.0]	3.0 [3.0-4.0]	
Fatigue				1
N experienced	74 (50.7)	32 (45.1)	42 (56.0)	
N experienced severe	29 (39.2)	13 (40.6)	16 (38.1)	
Severity	3.0 [2.0–4.0]	3.0 [2.0–4.0]	3.0 [2.0–5.0]	

*The earliest completed survey from 6, 9, or 12 months was included. Data are presented as frequency (column percentage) or median [25th to 75th percentile] with (minimum, maximum), as appropriate. Column percentage for those who experienced severe symptoms is the percentage of those who experienced the symptom. Number experienced severe symptoms are presented as column percentage of those who experienced the symptom
†Severity is scored 1 = not at all, 2 = a little, 3 = moderately, 4 = quite a bit, and 5 = extremely for only those who experienced a given symptom.
‡Missing symptoms were imputed to a "no" response. This imputation impacted 13 unique individuals.

Appendix 2 Table 5. Multivariable model results for primary and secondary outcomes comparing those positive for RSV during their hospital admission with those positive for COVID-19*

Outcome	Model Parameter Estimate (95% CI)
Primary Outcomes	
SF36 – Physical Functioning†	–0.09 (–6.37, 6.18)
EQ-5D-5L	–0.05 (–0.14, 0.03)
Self-Rated Health	0.18 (–5.78, 6.14)
Dyspnea‡	1.90 (1.14, 3.16)
Lawton ≥1 point decrease from baseline	1.08 (0.49, 2.38)
Katz ≥1 point decrease from baseline	1.00 (0.44, 2.26)
Secondary Outcomes	
PROMIS Sleep Disturbance ≥1SD	1.29 (0.67, 2.47)
PROMIS Cognitive Function ≥1SD	1.17 (0.54, 2.54)
PROMIS Social Activities ≥1SD	1.63 (0.83, 3.20)
CAP-Sym Total Score	1.12 (-2.09, 4.33)

*Models Includes predictor of interest COVID-19 (reference) vs. RSV, age, sex, race/ethnicity, smoking status, baseline functional limitations, and number of organ systems affected by chronic disease. For outcome models with baseline data available (Katz, Lawton, and SF36-PF), the matching retrospective baseline variable was included.

†Data are presented as beta coefficient (95% confidence interval) for those with RSV compared to the reference of COVID-19; multivariable linear regression models were used to compute these results.

‡Data are presented as odds ratios (95% confidence interval) for those with RSV compared to the reference of COVID-19; multivariable logistic regression with Firth correction for rare outcomes were used to compute these results as appropriate. Dyspnea results were computed using ordinal regression.

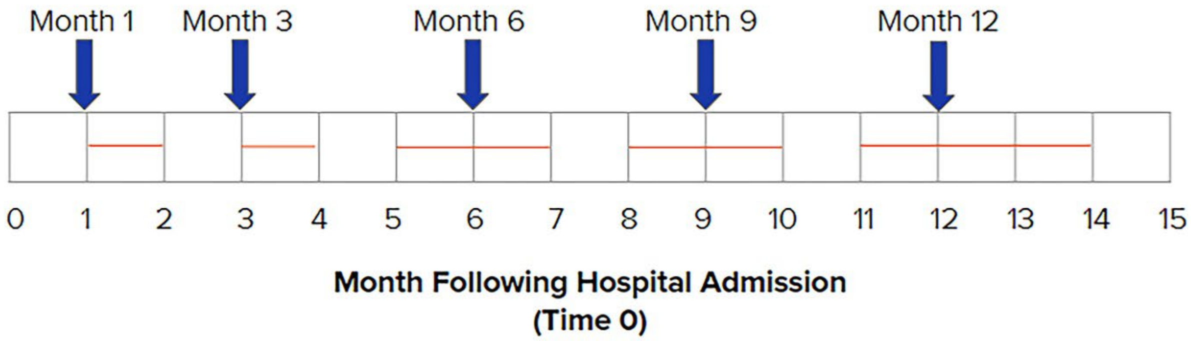

Appendix 2 Figure 1. Illustration of study survey timepoints for the current analysis (blue arrows) and full eligibility windows for each survey (orange lines). Timing for eligibility of each survey window is based on the date of hospital admission. Only the earliest survey from months 6, 9, and 12 were included in the current analysis.

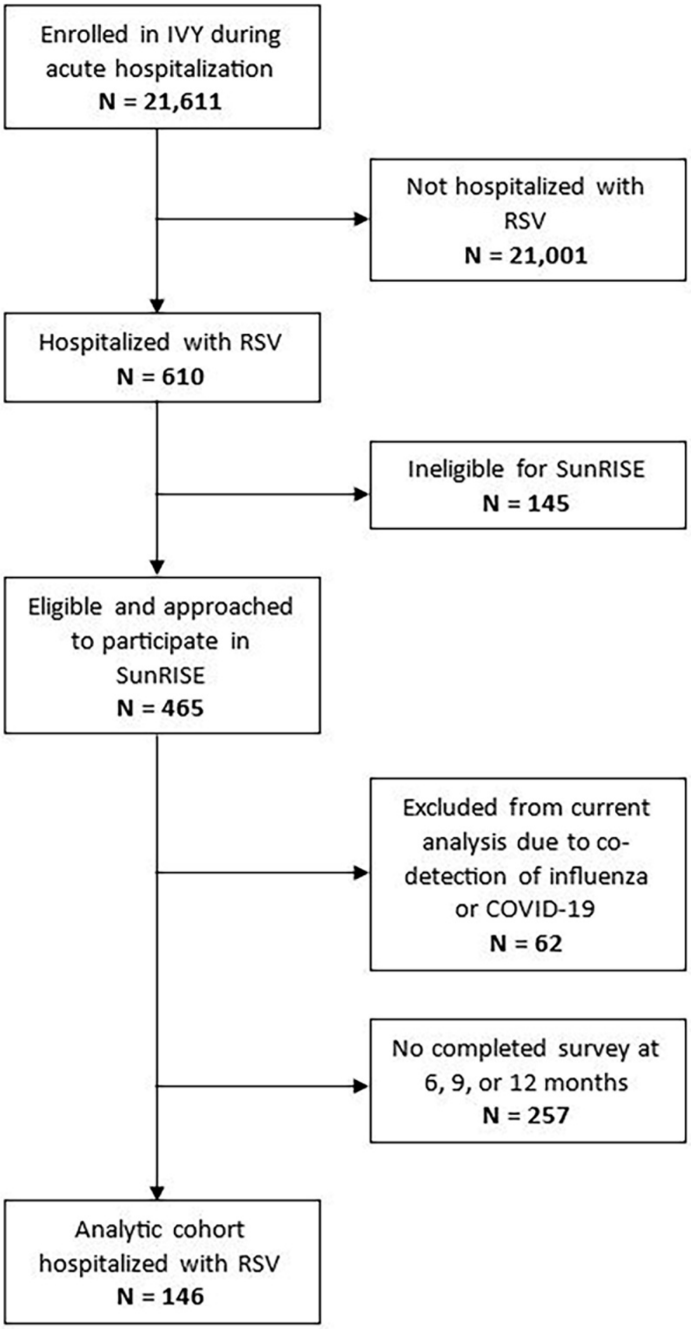

Appendix 2 Figure 2. Flow diagram for the creation of the final analytic cohort for those hospitalized with RSV participating in SunRISE.
